# Supplementary material for: Plasma Levels of Pentraxin 3: A Potential Prognostic Biomarker in Urinary Bladder Cancer Patients
Source: Int J Mol Sci. 2024 Mar 20;25(6):3473. doi: 10.3390/ijms25063473 (PMC10970929; doi:10.3390/ijms25063473)
Supplement: Supplementary file 1 [file ijms-25-03473-s001.zip › ijms-2912789-supplementary.pdf]

Supplementary Table S1. Pentraxin 3 (PTX3) levels in plasma and tumor characteristics.

	**PTX3** (ng/mL) Median (min-max)
**Invasiveness, n (%)**	
NMIBC	0.52 (0.07 – 3.72)
MIBC	1.08 (0.12-12.51)
**Metastatic disease, n (%)**	
No	0.73 (0.07-7.33)
Developed metastatic disease*	1.02 (0.37-12.51)
Primary metastatic disease	1.35 (0.50-7.33)
Missing	
**Tumor grade, n (%)**	
Grade 1	0.47 (0.24-3.72)
Grade 2	0.81 (0.16-3.99)
Grade 3	0.87 (0.07-7.33)
Missing (CIS)	
Low grade	0.51 (0.16-3.99)
High grade	0.87 (0.07-7.33)
Missing (CIS)	
**Histological subtype, n (%)**	
Urothelial carcinoma	0.74 (0.07-7.10)
UC with Squamous cell features	1.05 (0.28-12.51)
UC with Sarcomatoid features	1.4 (0.91-7.33)

NMIBC – Non-muscle invasive bladder cancer. MIBC – Muscle-invasive bladder cancer. CIS – Carcinoma In Situ. UC – Urothelial carcinoma. * Developed metastatic disease during follow-up. Primary metastatic disease – lymph node metastases found during cystectomy.
